# Supplementary material for: Assigning function to natural allelic variation via dynamic modeling of gene network induction
Source: Mol Syst Biol. 2018 Jan 15;14(1):e7803. doi: 10.15252/msb.20177803 (PMC5787706; doi:10.15252/msb.20177803)
Supplement: Supplementary file 1 — Appendix [file MSB-14-e7803-s001.pdf]

APPENDIX

Assigning function to natural allelic variation via dynamic modeling of gene network induction

Magali Richard[1,2], Florent Chuffart[1], Hélène Duplus-Bottin[1], Fanny Pouyet[1], Martin Spichty[1], Etienne Fulcrand[1], Marianne Entrevan[1], Audrey Barthelaix[1], Michael Springer[3], Daniel Jost[2] and Gaël Yvert[1]

1) Laboratoire de Biologie et de Modélisation de la Cellule, Ecole Normale Supérieure de Lyon, CNRS, Université Lyon 1, Université de Lyon; 69007 Lyon; France.

2) Univ Grenoble Alpes, CNRS, TIMC-IMAG, F38000 Grenoble, France.

3) Department of Systems Biology, Harvard Medical School, Boston, MA 02115, USA.

TABLE OF CONTENTS

Appendix Text S1. Description and analysis of the computational model of the GAL network.. p1-6
Appendix Table S1.. p7
Appendix Table S2.. p8

Appendix Text S2. Methods for molecular dynamics simulations..... p9-11

Appendix Figure S1.. p13
Appendix Figure S2.. p14
Appendix Figure S3.. p15
Appendix Figure S4.. p16
Appendix Figure S5.. p17
Appendix Figure S6.. p18
Appendix Figure S7.. p19
Appendix Figure S8.. p20
Appendix Figure S9.. p21
Appendix Figure S10.. p22
Appendix Figure S11.. p23
Appendix Figure S12.. p24

Appendix Table S3.. p25
Appendix Table S4.. p26

References.. p27

APPENDIX TEXT S1

MODELING THE GALACTOSE INDUCTION NETWORK

I. MODELING THE GALACTOSE INDUCTION NETWORK

We consider the model of the galactose induction network described in Fig.2a of the main text. For each gene we account for the status of the promoter (ON/OFF) and for the production and degradation of mRNAs and proteins. In addition, for the reporter gene, we account for the maturation of the fluorescent protein. We note P_1, P_3, P_{80} (resp. M_1, M_3, M_{80}) the level of proteins (resp. mRNAs). For the reporter gene, we note P_r (resp. P_r^*) the level of unmatured (resp. matured) proteins and M_r the level of mRNAs. The status of each promoter is described by the variables g_1, g_3, g_{80} and g_r. If gene x is OFF (resp. ON), $g_x = 0$ (resp. 1).

A. Derivation of the promoter ON/OFF switching rates

The status of the promoters is controlled by binding/unbinding event of Gal4, Gal80, Gal1 or Gal3 complexes. Gal4-dimers bind endogenously strongly to specific sequences, the so-called UAS sites, present in the GAL promoters to activate transcription (Giniger et al, 1988) . We assume that the endogenous level of Gal4 is important enough to maintain a constant occupation of UAS sites along the experiments, hence neglecting the effect of Gal4 variation on the induction of the network.

In absence of galactose, binding of Gal80-dimers to UAS sites leads to switch OFF the promoter state (Melcher et al, 2001). The regulatory function of Gal80 is performed via its homodimerized form (Pilauri et al, 2005). Under the fast equilibrium hypothesis, the level of Gal80 dimer is given by $P_{80}^d = (P_{80})^2/K_{d,80}$. Assuming cooperative binding of the dimers to the UAS sites, the promoter switching rate from ON to OFF for gene x is then given by $k_{off,x} = k_{off,x}^0 (P_{80}^d)^{n_x}$ with $k_{off,x}^0$ the association/binding rate and n_x the number of high-affinity Gal4p binding (UAS) sites, leading to

$$k_{off,x} = k_{off,x}^0 \left(\frac{P_{80}^2}{K_{d,80}}\right)^{n_x} \quad (1)$$

$$\equiv k_{off,x}^0 \left(\left[\frac{P_{80}}{K_{80}}\right]^2\right)^{n_x} \quad (2)$$

with $K_{80} \equiv \sqrt{K_{d,80}}$ an effective constant.

If present within the cell, galactose may bind to Gal1 and Gal3 proteins (Lavy et al, 2012). These "activated" molecules may form homodimers that may interact with Gal80-dimers at the GAL promoters (Timson et al 2002; Lavy et al, 2012), leading to the unbinding of Gal-80 to the UAS sites and to switch ON the promoter state. External galactose enters into the cell with the help of permeases. Among them is Gal2, a GAL-family gene under galactose-control (Tschopp et al, 1986). Since mutation of Gal2 does not seem to affect significantly the network response (Venturelli et al 2012), we decide, as in many other models (Acar et al, 2005; Venturelli et al, 2012; Apostu et al, 2012; Song et al, 2015), to also neglect the effect of Gal2 on the induction of the network. Hence, if we note $[gal]$ the concentration of galactose outside the cell and if we assume fast transport of galactose inside the cell, the intra-cellular concentration of galactose $[gal]_i$ would be considered as constant and directly proportional to $[gal]$: $[gal]_i \equiv f_i[gal]$. Internal galactose may bind to Gal3 protein to "activate" them (Lavy et al, 2012). Assuming, fast equilibrium between the active and normal forms of Gal3, this implies that the level of active proteins P_3^a is directly proportional to the total level P_3: $P_3^a = (k_{na,3}[gal]_i)/(k_{an,3} + k_{na,3}[gal]_i)P_3$ with $k_{na,3}$ (resp. $k_{an,3}$) the binding (resp. unbinding) rate of galactose to the normal (resp. active) form of Gal3. Noting $k_{an,3}/(f_i k_{na,3}) = K_{gal}$ the effective galactose dissociation constant, we have

$$P_3^a = \left[\frac{([gal]/K_{gal})}{1 + ([gal]/K_{gal})}\right] P_3 \quad (3)$$

It has been shown that the regulatory function of Gal3 is performed via homodimers of the active proteins (Lavy et al, 2012). Assuming again fast equilibrium between the monomers and the dimers implies that the level of Gal3 active dimers is given by $P_3^d = (P_3^a)^2/K_{d,3}$ with $K_{d,3}$ the dissociation constant of the dimer. Since Gal1 is a paralog of Gal3, we assume that it functions similarly as Gal3 via galactose-activated homodimers (Timson et al, 2002). Under the same hypothesis, we can therefore write that the level of active Gal1 dimers is given by $P_1^d = (P_1^a)^2/K_{d,1}$ with $P_1^a = ([gal]/K_{gal,1})/(1 + ([gal]/K_{gal,1}))P_1$.

Promoters switch from (Gal80-bound) OFF state to ON state by dissociation of Gal80-dimers from the UAS sites: this dissociation could be random (with rate $k_{on,x}^0$) or triggered by Gal1 or Gal3 dimers (with rates $k_{on,x}^1$ and $k_{on,x}^3$

respectively). This leads to a total OFF to ON promoter switching rate

$$k_{on,x} = k^0_{on,x}\left[1+\left(\frac{P^a_1}{K_1}\right)^2+\left(\frac{P^a_3}{K_3}\right)^2\right]^{n_x} \quad (4)$$

with $K_1 \equiv \sqrt{K_{d,1}k^0_{on,x}/k^1_{on,x}}$ and $K_3 \equiv \sqrt{K_{d,3}k^0_{on,x}/k^3_{on,x}}$, effective constants controlling the effect of Gal1 and Gal3 on the OFF to ON rate. The promoter status (g_x) may then evolve under both switching rates: $k_{off,x}$ (from $g_x = 1$ to $g_x = 0$, Eq.2) and $k_{on,x}$ (from $g_x = 0$ to $g_x = 1$, , Eq.13) that depends on the external galactose concentration $[gal]$ and of the current levels of Gal1 (P_1), Gal3 (P_3) and Gal80 (P_{80}) proteins.

B. Evolution of the mRNA and protein levels

We assume that the levels of mRNAs and proteins are determined by the balance between production (transcription for mRNAs, translation for proteins) and decay (degradation or dilution). The set of biochemical reactions driving this balance is given by

- The transcription rate of gene x ($x \in \{1, 3, 80, r\}$) is given by $\alpha_x + \Delta\alpha_x g_x$ with α_x the leaky transcription rate in the OFF state and $\alpha_x + \Delta\alpha_x$ the full transcription rate in the ON state.
- The propensity for mRNA degradation of gene x is given by $\beta_x M_x$ with β_x the mRNA degradation rate.
- mRNA translation into proteins is described by $\gamma_x M_x$ with γ_x the translation rate.
- The propensity for protein decay is given by $\mu_x P_x$ with μ_x the protein decay rate.
- For the reporter gene, we account for the maturation of newly created protein (P_r) into fluorescent ones (P^*_r). The corresponding propensity is given by $\delta_r P_r$ with δ_r the maturation rate.

C. Stochastic simulations

For a given set of parameters, the stochastic dynamics of galactose induction was simulated using a standard Gillespie algorithm (Gillespie et al, 1977). We generated 5,000 trajectories describing the stochastic time evolution of variables P_1, P_3, P_{80}, P_r, P^*_r, M_1, M_3, M_{80}, M_r, g_1, g_3, g_{80} and g_r. The system was first allowed to reach steady-state in absence of galactose ($[gal] = 0$) during 1,000 minutes. At $t = 0$, galactose was introduced and the induction response was simulated during 250 minutes of real time. At different time steps t along the trajectory, we monitored the current value of the fluorescence F defined as being proportional to the number of matured proteins plus the contribution of autofluorescence $F(t) = AP^*_r(t) + F_{auto}$ with A a constant and F_{auto} a random variable accounting for autofluorescence. Autofluorescence was modeled as a delta-correlated noise sampled from a log-normal distribution $F_{auto} = B\exp[Cz]$ with B and C two constants defining the mean ($B\exp[C^2/2]$) and variance ($B^2\exp[C^2](\exp[C^2]-1)$) of the distribution, and z a standard normal variable sampled from a normal distribution (mean=0, variance=1). As for the experiments (see main text), we defined the simulated cell as OFF (resp. ON) if $F(t)$ is below (resp. above) a given threshold thr. To model extrinsic noise (fluctuation of parameters between different single cells), in each simulated cell, model parameters y were initially drawn from a log-normal distribution and then kept constant along the whole simulated trajectory ($y = \bar{y}\exp[\sigma z]$).

D. Parameter values

Most of the parameters (except K_3, K_1, K_{80} and K_{gal}) used in the model have been fixed based on the literature and our own experiments and calculations (values are summarized in Table S1):

- mRNA life-time τ_m of some GAL genes have been recently measured by Hsu et al (Hsu et al, 2012) ($\sim$ 6min for GAL1 and $\sim$ 2 min for GAL3). From this, we estimated the degradation rate $\beta_x = \log 2/\tau_m$. For GAL80, we assumed $\beta_{80} = \beta_3$.

- For the GAL proteins, we assumed that their life-times are higher than the cell doubling time T. Therefore, the GAL protein decay rate is supposed to be identical for all GAL genes and equal to the dilution rate defined as $\mu_x = \mu = \log(2)/T$. Measurement of growth curves in exponential phase for different strains used in the study (Table S2) shows that the doubling time is about $T = 140$ minutes ($\mu = 0.005\text{min}^{-1}$) and does not depend significantly on the investigated strain, ie replacement of GAL3 alleles does not perturb growth during the duration of the experiments.

- For the reporter gene, we used a GFP protein (EGFP3-Cln2pest) with rapid maturation time and fast turnover. The maturation time is about 10 min (Iizuka et al, 2011) ($\delta_r = 0.07\text{min}^{-1}$) , the protein life-time is about 30 minutes (Mateus et al, 2000) ($\mu_r = 0.023\text{min}^{-1}$) and the mRNA life-time is about 10 minutes (Lin et al, 2016) ($\beta_r = 0.07\text{min}^{-1}$). Since the reporter gene is controlled by a GAL1 promoter, we assumed that $\alpha_r = \alpha_1$ and $\Delta\alpha_r = \Delta\alpha_1$.

- For the translation rates and the association and dissociation rates of Gal80 dimers to promoters, we assume, for simplicity, that they do not depend on the gene ($\gamma_x \equiv \gamma$, $k^0_{on,x} \equiv k^0_{on}$ and $k^0_{off,x} \equiv k^0_{off}$) and are equal to standard values: $\gamma = 1$ protein/mRNA/min and $k^0_{on} = k^0_{off} = 0.5\text{min}^{-1}$ (Milo et al, 2010).

- At full galactose induction (time=250min), we measure experimentally that the squared coefficient of variation CV^2 (defined as the ratio between the variance and the mean squared of the distribution of fluorescence) is equal to 0.25. We use the phenomenological relation found by Bar-Even et al (Bar-Even et al, 2006) relating CV^2 to the mean protein abundance $\langle p\rangle$ in yeast cell ($CV^2 \approx 1200/\langle p\rangle$) to estimate the number of matured fluorescent proteins in our experiments at full induction, $\langle P^*_r\rangle \approx 5,000$ molecules. Using previously defined values for the translation, degradation and maturation rates, this implies that $\alpha_1 + \Delta\alpha_1 \approx 0.132$. Hsu et al in (Hsu et al, 2012) showed that there is a 500-fold enrichment of the Gal1 mRNA level after galactose induction, ie $(\alpha_1 + \Delta\alpha_1)/\alpha_1 \approx 500$. This leads to $\alpha_1 = 0.021$ mRNA/min and $\Delta\alpha_1 = 10.6$ mRNA/min. Previous experimental attempts to quantify the relative number of Gal proteins in single cells have shown that at full galactose induction, the number of Gal1p is in average about 10 times higher than the numbers of Gal3p or Gal80p (Gash et al, 2000; Paulo et al, 2015), ie $\langle P_3\rangle \approx \langle P_{80}\rangle \approx \langle P_1\rangle/10$ at full induction. As for GAL1, knowing from Hsu et al that $(\alpha_3 + \Delta\alpha_3)/\alpha_3 \approx 12$ and $(\alpha_{80} + \Delta\alpha_{80})/\alpha_{80} \approx 5$ (Hsu et al, 2012), we can estimate that $\alpha_3 = 0.22$ mRNA/min, $\Delta\alpha_3 = 2.56$ mRNA/min, $\alpha_{80} = 0.45$ mRNA/min and $\Delta\alpha_{80} = 1.76$ mRNA/min.

- The number of high-affinity Gal4p-binding sites depends on the GAL promoter (Giniger et al, 1988; Venturelli et al, 2012; Hsu et al, 2012). GAL3 and GAL80 promoters contain one single site ($n_3 = n_{80} = 1$) while GAL1 promoter has at least two high-affinity sites ($n_1 = n_r = 2$).

- Autofluorescence parameters B and C (see above) were fixed by measuring the mean and variance of the fluorescence for a strain that does not carry the reporter gene. We found $B = 6.55$ and $C = 0.365$. The constant factor A that relate the number of matured fluorescent proteins to the actual fluorescence was adjusted to match the experimental mean fluorescence at full induction, $A = 0.016$. For the threshold thr, we use the experimentally defined value 23.

Note that all these parameter estimations represent the average values of log-normal distributions from which we drawn a random set of parameter at the beginning of every simulation to model extrinsic noise (see above). The strength of extrinsic noise was adjusted by varying σ in order that the CV^2 predicted by the model from the distribution of fluorescence at full induction (time=250min) matched the experimental value. This leads to $\sigma = 0.125$.

Strains used to study the diauxic shift (see main text) are from a different background (s288c) and contains a different reporter gene (YFP also under a GAL1 promoter control). For simplicity, for these strains, we used the same parameters except the reporter gene and fluorescence parameters: $\beta_r = 0.04\text{min}^{-1}$, $\delta_r = 0.017\text{min}^{-1}$, $\mu_r = \mu = 0.005\text{min}^{-1}$, $A = 11$, $B = 3.74$, $C = 0.365$ and $thr = 90$.

E. Analysis of the model

1. *Deterministic stability*

Before focusing on the kinetic stochastic activation of the pathway, we analyze the corresponding deterministic model at steady-state:

$$\frac{d\,g_x}{dt} = k_{on,x}(1 - g_x) - k_{off,x}g_x = 0 \tag{5}$$

$$\frac{d\,M_x}{dt} = \alpha_x + \Delta\alpha_x g_x - \beta_x M_x = 0 \quad (6)$$

$$\frac{d\,P_x}{dt} = \gamma_x M_x - \mu_x P_x = 0 \quad (7)$$

To simplify, we note P'_x the steady-state value of GALx proteins P_x normalized by its value in absence of galactose and in feedback loops $\alpha_x\gamma_x/(\beta_x\mu_x)$. Substitutions in Eqs.(5-7) leads to the following set of equations that determines P'_x:

$$P'_1 = 1 + \left(\frac{\Delta\alpha_1}{\alpha_1}\right) \frac{1}{1 + \left[\left(\frac{\Delta\alpha_3}{\alpha_3}\right)\frac{1}{(P'_3-1)} - 1\right]^2} \quad (8)$$

$$P'_{80} = 1 + \left(\frac{\Delta\alpha_{80}}{\alpha_{80}}\right)\left(\frac{\alpha_3}{\Delta\alpha_3}\right)(P'_3 - 1) \quad (9)$$

$$P'_3 = 1 + \left(\frac{\alpha_3}{\Delta\alpha_3}\right) \frac{1}{1 + \frac{\left(P'_{80}\rho_{Gal80}\right)^2}{1+\left(P'_1\rho_{Gal1}\epsilon\right)^2+\left(P'_3\rho_{Gal3}\epsilon\right)^2}} \quad (10)$$

with $\epsilon = ([gal]/K_{gal})/(1 + ([gal]/K_{gal}))$ defining the action of galactose and $\rho_{Galx} \equiv (\alpha_x\gamma_x)/(\beta_x\mu_x K_x)$, the so-called strength of GALx. The deterministic stability of the system is therefore given by solving this system of equations. Depending on the parameters, such systems have one unique solution (monostability) or two stable solutions (bistability). Eqs. (8-10) suggests that the stability of the system is determined by only 7 effective parameters (ρ_{Gal1}, ρ_{Gal3}, ρ_{Gal80}, $K_{gal}/[gal]$, $\Delta\alpha_1/\alpha_1$,$\Delta\alpha_3/\alpha_3$,$\Delta\alpha_{80}/\alpha_{80}$). In Fig.4d, we plot bifurcation diagrams in the (ρ_{Gal3}, K_{gal})-space for different values of $[gal]$. The diagram is partitioned into 3 regions: one monostable "OFF" region where the effect of galactose or the strength of positive feedback loops are not strong enough to activate the transcriptional response of the network, one monostable "ON" region where the response is activated, and one bistable region where both ON and OFF responses are (deterministically) stable.

2. Stochastic induction response

Similarly to the deterministic stability, we expect that phenotypic - experimentally measured - behavior (the evolution of the fraction of ON cells as a function of time after galactose induction) is mainly controlled by few effective parameters.

The promoter switching rate from OFF to ON is driven by the current number of activated Gal1p (P_1^a) and Gal3p (P_3^a) normalized by effective constants (K_1 and K_3). Therefore, we hypothesized that induction of the network would depend on the ratios between the average number of activated molecules just after the introduction of galactose and the effective constants, ie $(\alpha_1\gamma_1)/(\beta_1\mu_1K_1)([gal]/K_{gal})/(1+([gal]/K_{gal}))$ and $(\alpha_3\gamma_3)/(\beta_3\mu_3K_3)([gal]/K_{gal})/(1+([gal]/K_{gal}))$. This suggests that induction would be controlled by the values of ρ_{Gal1}, ρ_{Gal3}, K_{gal} and $[gal]$. Identically, the promoter switching rate from ON to OFF is driven by the current number of Gal80p normalized by K_{80}. The decision to remain OFF after galactose introduction would therefore depend on the strength of Gal80 ρ_{Gal80}.

The kinetic behavior of the model would thus be mainly determined by the competition between the repressing effect of Gal80 (controlled by ρ_{Gal80}) and the activating role of Gal1 and Gal3 (controlled by ρ_{Gal1}, ρ_{Gal3} and the ratio $[gal]/K_{gal}$). Strong activation (compared to repression) would lead to fast transitions from OFF to ON and a gradual response (Fig.S3a,b and Fig.S5). On contrary, weak activation (weak ρ_{Gal1}, ρ_{Gal3} values and/or low galactose concentration) leads to delayed transitions and a binary response (Fig.S3 a,b and Fig.S5). Interestingly when the strength of Gal1 is about the same magnitude of ρ_{Gal80}, the effect of Gal3 is very limited on the inducibility (Fig.S3 c).

For fixed values of $[gal]$ and K_{gal}, we verified that the inducibility mainly depends on the values of ρ_{Gal1}, ρ_{Gal3} and ρ_{Gal80} and not significantly on the precise values of parameters composing them (Fig.S4 a,b,c). Then, we checked that, on contrary, inducibility does not significantly depend on other parameters like the full transcription rates ($\alpha_x + \Delta\alpha_x$) (Fig.S4 a,b,c). When the system becomes more stochastic (weak galactose concentration or ρ_{Gal1}, ρ_{Gal3}), changing $\Delta\alpha_x$ when keeping ρ_{Galx} fixed starts to have more important effect on the inducibility (Fig.S4 d). However, this effect is hard to dissociate from a change in ρ_{Galx} itself.

We verified using *in silico* simulations, that the binary-vs-gradual induction of pGal1-GFP also corresponded to binary-vs-gradual induction of Gal1p, Gal3p and Gal80p (Fig. S12). In addition, the model predicts that choosing GFP proteins with a longer life time (equal to the cell doubling time, such as for YFP) would also provide a suitable - yet less precise- characterization of the global network kinetics (Fig.S12).

3. Specific role of ATP binding

In addition to galactose, active Gal3 and Gal1 proteins are bound to ATP (Lavy et al, 2012). In the model described above, we assumed fast and efficient ATP binding and did not explicitly account for it. In Section "Linking GAL3 alleles to specific parameter values" of the main text, we studied the effect of point-mutations within the ATP pocket (W177A and W117T). In this part, we derive new expressions for K_{gal} and ρ_{Galx} integrating ATP binding. We consider that activation of Gal1p and Gal3p occurs in two steps: binding of galactose and binding of ATP.

If ATP binds first then the fast equilibrium hypothesis applied to the fully-active, pre-activated (bound only to ATP) and unbound forms of Gal3p (idem for Gal1p) leads to a level of active proteins

$$P_3^a = \left(\frac{([gal]/K'_{gal})}{1 + ([gal]/K'_{gal})} \right) P_3 \quad (11)$$

with $K'_{gal} = K_{gal}(1 + k_u/(k_b[ATP]))$ a renormalized effective galactose dissociation constant that depends on binding (k_b) and unbinding (k_u) rates of ATP. In this case, accounting explicitly for ATP binding does not modify the structure of the model but leads to renormalize K_{gal}. Therefore, a deleterious mutation within the ATP pocket will destabilize ATP binding leading to a smaller ratio $r_{ATP} \equiv k_b[ATP]/k_u$ and $K'_{gal} = K_{gal}(1 + 1/r_{ATP})$ will increase compared to the wild-type -not mutated - case.

If galactose binds first (which is more likely given that it is buried deeper than ATP within the structure of activated Gal3p (Lavy et al, 2012)), the fast equilibrium hypothesis applied to the fully-active, pre-activated (bound only to galactose) and unbound forms of Gal3p (idem for Gal1p) leads to a level of active proteins

$$P_3^a = \left(\frac{k_b[ATP]}{k_u + k_b[ATP]} \right) \left(\frac{([gal]/K'_{gal})}{1 + ([gal]/K'_{gal})} \right) P_3 \quad (12)$$

with $K'_{gal} = K_{gal} k_u/(k_u + k_b[ATP])$ a renormalized effective galactose dissociation constant. This leads to a total OFF to ON promoter switching rate

$$k_{on,x} = k^0_{on,x} \left[1 + \left(\frac{P_1 \epsilon'}{K'_1} \right)^2 + \left(\frac{P_3 \epsilon'}{K'_3} \right)^2 \right]^{n_x} \quad (13)$$

with $\epsilon' = ([gal]/K'_{gal})/(1 + ([gal]/K'_{gal}))$ and $K'_3 \equiv K_3(k_u + k_b[ATP])/(k_b[ATP])$ (idem for K'_1) a renormalized effective constant controlling the effect of Gal3 on the OFF to ON rate. Therefore, in this case, accounting explicitly for ATP binding also does not modify the structure of the model but leads to renormalize the 2 key effective parameters that drive the stochastic induction response K'_{gal} and ρ'_{Gal3} (via the renormalization of K_3). Similarly, our model predicts that for a deleterious mutation within the ATP pocket, $K'_{gal} = K_{gal}/(1 + r_{ATP})$ will increase while $\rho' = \rho[r_{ATP}/(1 + r_{ATP})]$ will decrease compared to wild-type. Moreover, since

$$\left[\frac{K'_{gal}(mutant)}{K'_{gal}(wt)} \right] \times \left[\frac{\rho'_{gal}(mutant)}{\rho'_{gal}(wt)} \right] = \left(\frac{r_{ATP}(mut.)}{r_{ATP}(wt)} \right) \left(\frac{1 + r_{ATP}(wt)}{1 + r_{ATP}(mut.)} \right)^2 > 1 \quad \text{for } r_{ATP}(mut.) < r_{ATP}(wt) \quad (14)$$

the model further predicts that the impact of the deleterious mutation will be stronger on K'_{gal} than on ρ'.

F. Parameter inference

In our work, we study nearly-isogenic strains where only the GAL3 allele was different. Therefore, we expect that phenotypic differences between these strains arise mainly from modifications of ρ_{Gal3} and K_{gal} (we neglect the effect on $\Delta\alpha_3$).

To infer the allele-dependency of ρ_{Gal3} and K_{gal}, we first arbitrarily fixed the values of ρ_{Gal1} and ρ_{Gal80} and then compute the inducibility for several values of ρ_{Gal3} (by modifying K_3) and K_{gal} at the three investigated galactose concentrations ($[gal] = 0.05\%$, 0.1% and 0.5%). Parameters were sampled from a 2D grid encompassing the region of interest using a logarithmic scale: $(\rho_{Gal3}, K_{gal}) = (\rho^o_{Gal3} \times 10^{0.05i}, K^o_{gal} \times 10^{0.05j})$ (for $i \in \{-10, 10\}$ and $j \in \{-10, 10\}$). Finally, for each strain, we found the set of parameters that minimized the global chi2-score between the measured and predicted fractions R of ON cells at different time points after induction and for the different galactose concentrations: $S = \sum_{[gal]} \sum \text{time}_{\ t_i} (R_{exp}(t_i) - R_{pred}(t_i))^2$. Uncertainties on the parameters (Fig.4a,b of the main text) reflect the size of the sampling grid around the optimal parameters.

Parameter inference was repeated 6 times for different values of ρ_{Gal1} and ρ_{Gal80} (by modifying K_1 and K_{80}) $[(\rho_{Gal1}, \rho_{Gal80}) = (50, 250), (100, 250), (50, 200), (100, 200), (50, 150), (25, 100)]$. Note that if ρ_{Gal1} is too strong (about the level of ρ_{Gal80} or beyond), the model cannot reproduce the diversity of response observed experimentally for the different strains (Fig.S3 c). In particular, inducibilities of more stochastic strains cannot be well fitted. That is why we focus on ρ_{Gal1} values well below ρ_{Gal80}.

Inferred parameters given in Fig.3a of the main text and in Fig. S6, S7 and S9 was computed for $(\rho_{Gal1}, \rho_{Gal80}) = (100, 250)$.

It is interesting to note that, for the galactose concentrations investigated in our study ($[gal] = 0.05, 0.1, 0.5\%$), all strains mapped to the monostable ON region of the deterministic bifurcation diagram (Fig. 4d). This implies that, at these concentrations, the observed stochastic binary response of some strains is only transient and should later converge to a unimodal distribution peaked around the ON level of expression. This convergence may be very slow (for example for the GAL3(BY) allele at $[gal] = 0.05\%$, the model predicts a convergence time of 11h) and may not be achieved during the typical time windows (4 to 8 h) used in standard "steady-state" experimental setups.

description	param.	GAL1	GAL3	GAL80	reporter GFP	reporter YFP
leaky transcription rate	α_x (min^{-1})	0.021	0.22	0.45	0.021	0.021
diff. in transcription rate between full and leaky state	$\Delta\alpha_x$ (min^{-1})	10.6	2.56	1.76	10.6	10.6
number of high affinity Gal4p binding sites at promoter	n_i	2	1	1	2	2
mRNA degradation rate	β_x (min^{-1})	0.12	0.35	0.35	0.069	0.04
translation rate	γ_x (min^{-1})	1	1	1	1	1
protein decay rate	μ_x (min^{-1})	0.005	0.005	0.005	0.023	0.005
Gal80p association rate at promoter	$k^0_{on,x}$ (min^{-1})	0.5	0.5	0.5	0.5	0.5
Gal80p dissociation rate at promoter	$k^0_{off,x}$ (min^{-1})	0.5	0.5	0.5	0.5	0.5
effective galactose dissociation constant	$K_{gal,1}$ (%)	0.06	/	/	/	/
reporter gene maturation rate	δ_r (min^{-1})	/	/	/	0.069	0.017
protein to fluorescence conversion constant	A	/	/	/	0.016	11
autofluorescence parameter	B	/	/	/	6.55	3.74
autofluorescence parameter	C	/	/	/	0.365	0.365
ON/OFF decision threshold	thr	/	/	/	23	90

Appendix Table S. 1: Description of model parameters used in this study

**strain (GAL3 allele)**	T
GY1648 (BY)	139
GY1689 (NCYC361)	136
GY1692 (K11)	138
GY1695 (Y12)	136
GY1698 (DBVPG1788)	140
GY1704 (DBVPG1853)	135
GY1707 (YJM978)	137
GY1713 (JAY291)	134

Appendix Table S. 2: Cell doubling time T measured experimentally for the strains investigated in this study (in min)

APPENDIX TEXT S2
Methods for molecular dynamics simulations

Software: Model systems were set up with the program CHARMM (Brooks *et al*, 2009), version c39b1. Initial input files for CHARMM were generated with the CHARMM-GUI server (Lee *et al*, 2016) and then modified to implement the actual structural model with dual-topology (see below). Molecular dynamics simulations for free energy calculations were carried out with the program NAMD, version 2.11 (Phillips *et al*, 2005). Poisson-Boltzmann equations were carried out with the program ABPS (Baker *et al*, 2001), initial files were obtained from the PDB2PQR server (Dolinsky *et al*, 2004, 2). Visualizations were done with the program VMD (Humphrey *et al*, 1996).

Model system: The tetrameric Gal3p*-Gal80p complex has spacious dimensions of about 120x90x60 Å which is too large to achieve sufficient sampling by molecular dynamics in a reasonable time with adequate (explicit) solvation. We worked therefore with a structurally reduced model system (Appendix Figure S8A) to perform the alchemical double mutation (2 x Gal3p*-H352D) in the Gal3p* dimer and Gal3p*-Gal80p tetramer. Our goal is to determine how the mutation H352D changes the affinity between the activated conformation of Gal3p* and its binding partner Gal80p. For the two legs of the alchemical cycle depicted in Fig. 6e, the conformation of Gal3p* is therefore assumed to be identical in regions that are distant from the mutational sites. The calculation does not take into account the impact of the double mutation on global conformational changes that occurs when non-activated Gal3p binds to Gal80p. We further assume that distant regions of Gal80p and Gal3p* (including the galactose and ATP molecules) have little influence on the dynamics of the mutated residue 352 and its neighbors ($1/r^2$-dependence of long-range electrostatic forces). But the distant regions are important for stabilizing the overall complex structure, and they contribute to the free energy change $\Delta G_{alchemical}$ through direct long-range electrostatic interaction energies (1/r-dependence). In the case of distant residues of Gal3p*, these latter long-range contributions cancel to when subtracting the two alchemical legs of the thermodynamic cycle of Fig. 6e (assumption of similar 3D structures). We decided therefore to remove a relatively large part of distant Gal3p* residues. In contrast, the distant Gal80p residues contribute only in the case of the tetramer. We therefore removed a much smaller part of Gal80p. We verified that the removed Gal80p region was charge neutral so that its electrostatic contribution to $\Delta G_{alchemical}$ is minimal. To compensate for the loss of stabilization due to the removal of distant residues, we restrained the residues in proximity to the removed regions.
The actual model of the tetramer was constructed as follows: starting from the crystal structure of the tetrameric Gal3p*-Gal80p complex (PDB entry 3V2U), we replaced His352 of both Gal3p* monomers by a residue with a dual sidechain topology (Chipot & Pohorille, 2007) for histidine (with the proton on nitrogen ND1) and deprotonated aspartic acid. In addition to the non-resolved residues of the crystal structure, we neglected all Gal3p* residues that had no atom within 20 Å of neither residue 352 (of Gal3p*; the Cα atoms were used as center of the two perimeters). For the removal of Gal80p residues we used a perimeter of 35 Å. The N-terminal ends of truncated protein fragments were patched with acetyl groups; the C-terminal ends were capped with N-methyl groups. We harmonically restrained with a force constant of 100 kcal/ $Å^2$ all residues outside a perimeter of 15 Å around mutated

residues 352. The remaining proximate residues were allowed to move freely. This system was solvated by cubic box (side length of 90 Å) of TIP3 water molecules, chloride and potassium ions (ionic strength of 0.15 M). The entire system was charge neutral when residues 352 mimic aspartic acids, and +2*e* in the case of histidine topology. For the setup of the dimer the same procedure was followed but deleting the Gal80p dimer prior to solvation (using the same box size). The entire system was also charge neutral for Asp-topology or+2*e* for the His-topology.

Settings for molecular dynamics: The systems were simulated with the CHARMM36 force field and periodic boundary conditions. A cutoff of 12 Å was used for short range nonbonded interactions whereas long-range electrostatic interactions were treated by Particle-Mesh Ewald (PME) with a grid spacing of 1 Å. The equation of motion was integrated with a time step of 1 fs. Short range nonbonded forces were updated every 2 fs, PME forces every 4 fs. The pressure was kept constant at 1 atm with a piston oscillation period of 100 fs and a damping time scale of 50 fs. To accelerate the dynamics and thereby enhance sampling, we used an elevated temperature of 343 K instead of 303 K (= temperature of the experiments). Such an increase in temperature is supposed to decrease the dielectric constant of the water model. Because the dielectric constant of CHARMM's TIP3P model is about 25 % higher than the one of water at 310 K (Höchtl *et al*, 1998), the simulation at 343 K mimics actually more closely the dielectric constant of water at 310 K than at the simulation at 310 K.
With these MD settings the average box size of both systems (dimer and tetramer) was almost identical (90.7±0.1 *vs*. 90.5±0.1 Å) in the following free energy calculations. For systems with identical (cubic) box sizes and identical changes in net charge, finite-size effects should largely cancel when comparing the two legs of the alchemical mutations (Hummer *et al*, 1996; Figueirido *et al*, 1997).

Alchemical free energy calculations: For the alchemical mutations we used a standard dual topology approach (Chipot & Pohorille, 2007) where the two residues 352 of Gal3p were transformed from Asp to His as a function of the usual coupling parameter λ. The reader is referred to the literature for more details (Chipot & Pohorille, 2007); here only the essence of alchemical free energy calculations is given. In a typical alchemical transformation, the nonbonded interactions of the outgoing residues (here 2 x Asp sidechains) with their environment are removed and those of the incoming residues (2 x His sidechains) added when the parameter λ is changed from 0 to 1. The free energy change is calculated for changing λ from 0 to 1. In our case the electrostatic interactions of the outgoing residues are linearly removed from λ=0 to λ=0.5 and those of the incoming residues linearly added from λ=0.5 to λ=1. (alchElecLambdaStart=0.5). The van-der-Waals interactions are scaled down from λ=0 to λ=1 for the outgoing residues, and scaled up from λ=0 to λ=1 for the incoming residues (alchVdWLambdaEnd=1). To avoid endpoint problems, we used NAMD's soft-core potentials with a shifting coefficient of 4 $Å^2$. Nonbonded interactions within outgoing and incoming atoms were also (de)coupled to account for the repulsive electrostatic interaction between the two charged Asp residues.
The actual transformation was done with a windowing method where the dimer and tetramer systems were sampled by molecular-dynamics at 21 equally-separated λ-values (0,0.05, 0.1, 0.15, 0.2, 0.25, 0.3, 0.35, 0.4, 0.45, 0.5, 0.55, 0.6,0.65, 0.7, 0.75, 0.8, 0.85, 0.9, 0.95, 1). For each window we performed two separate MD simulations and recorded on-the-fly with NAMD's FEP option every 100 fs the work required to switch instantaneously the Hamiltonian from the actual window to one of the neighboring windows; for the endpoints at λ=0 and 1 we performed only one MD simulation (because there is only a single neighbor).

For the simulation of the tetramer at λ=0 (resembling 2 x Asp) , we saved snapshots during the production run every 10 ps (Figure 6d shows an example).
The free energy difference between two neighboring windows was calculated from the collected work data for forward and reverse switches using Bennett's acceptance ratio (BAR) method (Bennett, 1976). The total free energy change for the transformation of λ from 0 to 1 was obtained by summing up all differences between neighboring windows. Because we defined $\Delta G_{alchemical}$ as the free energy difference for the alchemical transformation from His to Asp (see Figure 6e), the value of $\Delta G_{alchemical}$ actually corresponds to the change of λ from 1 to 0. After an equilibration phase of 1.5 ns in each window, we monitored for blocks of 100 ps the value of $\Delta G_{alchemical}$ (Appendix Figure S8B). A block size of 100 ps contains 2000 work values for forward and reverse switches between neighboring windows. The value of $\Delta G_{alchemical}$ fluctuates around a mean value of -228.3 ± 0.7 kcal/mol in the case of the tetramer and -225.5 ± 0.5 kcal/mol in the case of the dimer. The errors correspond to twice the standard error of the mean.
These free energy values seem too large at a first glance: Karplus and coworkers calculated for the alchemical *single* mutation Asn to Asp in a protein environment a value of -95.1 kcal/mol (Archontis *et al*, 1998). Thus, their value is about 20 kcal/mol less negative than half of our value for the *double* mutation in the tetramer. This large difference arises mainly due to the scaling of nonbonded interactions within the outgoing Asp residues (which was not required in the study of Karplus and coworkers).
Finally, the difference between the two legs of the thermodynamic cycle of Fig. 6e can be calculated: $\Delta G_{sub}^{D352} - D\Delta G_{sub}^{H352} = \Delta G_{alchemical}^{tetramer} - \Delta G_{alchemical}^{dimer} = 2.8 \pm 0.9$ kcal/mol; the usual error propagation rule was used for this subtraction.

Electrostatic potential: For the crystal structure of the tetrameric Gal3p*-Gal80p complex (PDB entry 3V2U) we calculated the electrostatic potential by solving the Poisson-Boltzmann equation with parameters of the CHARMM36 force field and applying standard PDB2PQR parameters. Non-resolved protein residues in the crystal structure and the cofactors (ATP, galactose) were neglected.

APPENDIX FIGURES

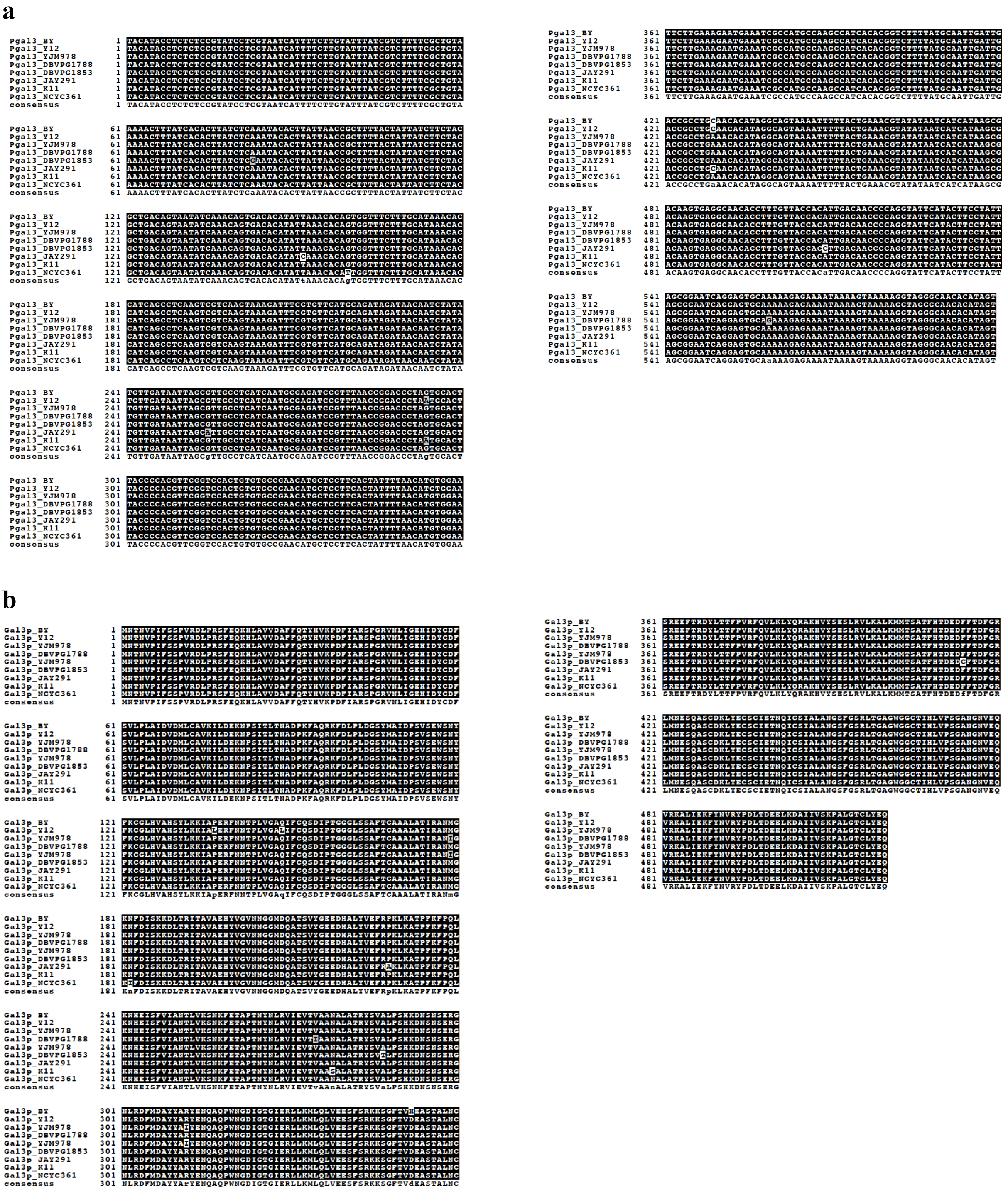

Appendix Figure S1. Sequences of natural *GAL3* locus used in this study. (**a**) Nucleotidic sequences of *GAL3* promoters. (**b**) Amino-acid sequences of Gal3p proteins. Alignment was performed using T-Coffee and visualized using Boxshade.

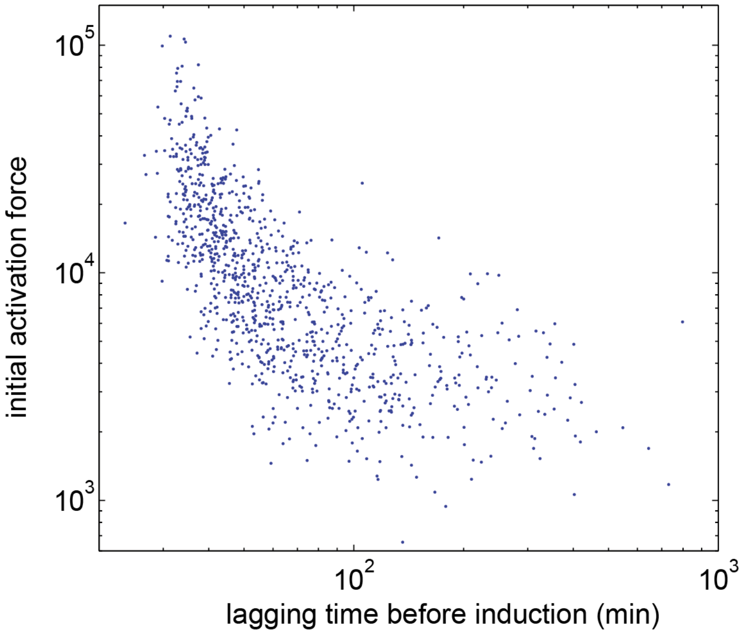

Appendix Figure S2. **The lagging time in binary system depends on the initial activation force.** For the same parameters as in Fig. 2c, from each single-cell trajectory, we estimate the lagging time before single-cell reaches the threshold distinguishing ON from OFF cells (dashed line in Fig. 2c). For 1000 simulated trajectories, we plot it as a function of the initial activation force defined as the value of the OFF to ON switching rate of GAL promoters k_i^{on} just at the moment of induction. This parameter depends on the initial number of Gal1p and Gal3p and on the effective constants K_1 and K_3 (see Materials and Methods and Appendix Text S1). The Spearman correlation between lagging time and initial activation force is -0.75.

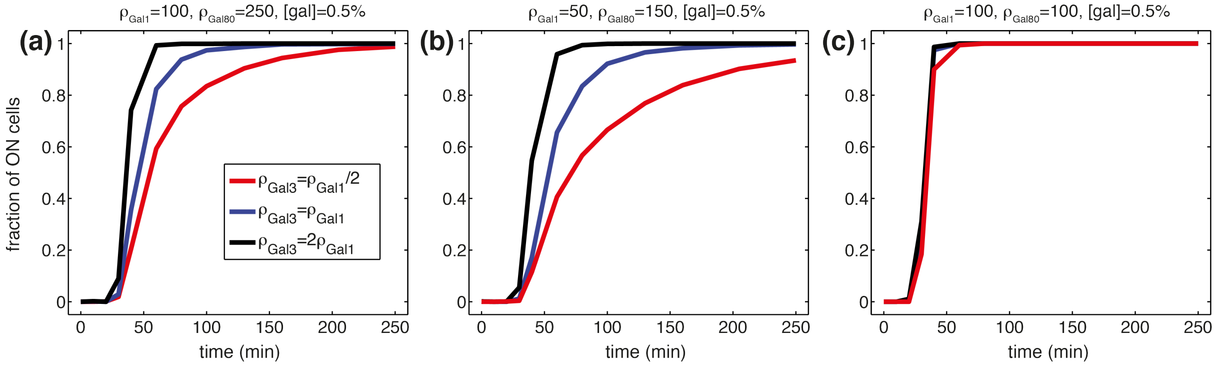

Appendix Figure S3. Effect of ρ_{Gal1} , ρ_{Gal3} and ρ_{Gal80} values on network inducibility. Each panel shows the induction of the network as a function of time for different values of ρ_{Gal3} (colored lines) in a specific context of ρ_{Gal1} and ρ_{Gal80} values. Galactose concentration and K_{gal} were fixed to *[gal]* = 0.5% and K_{gal} = 0.055%.

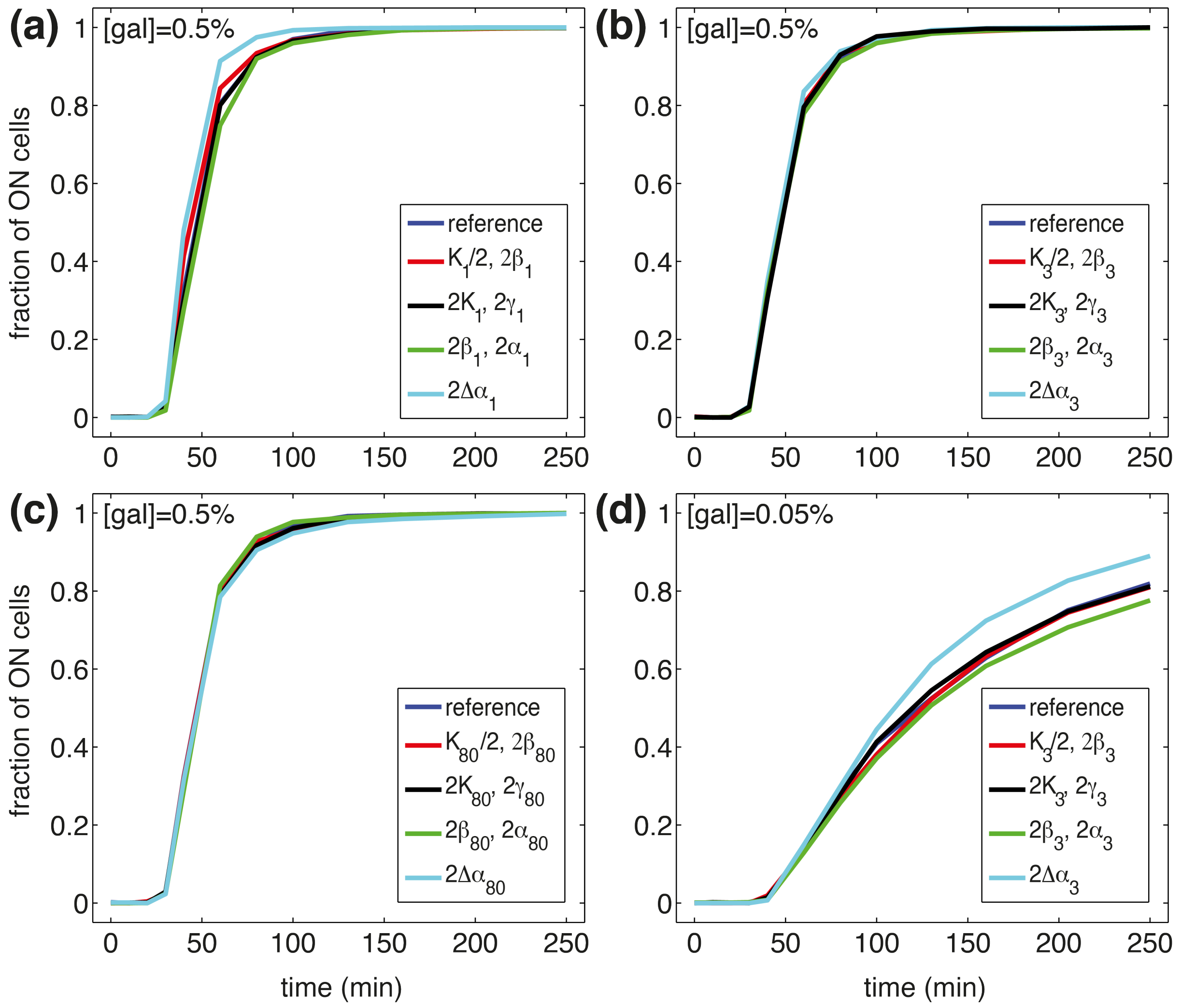

Appendix Figure S4. Inducibility predictions depend on ρ_{Gal1} , ρ_{Gal3} and ρ_{Gal80} meta-parameters rather than on their constituent parameters. Each panel represents model predictions of inducibility as a function of time after induction at the indicated galactose concentration. Colors correspond to different sets of parameter values in the model, blue (reference) referring to values of Appendix Table S1 completed with K_1=0.35, K_3=1.26, K_{80}=1.03 and K_{gal}=*0.055%*. (**a**) Parameters constituting ρ_{Gal1} (formula $\rho_{Gal1}=\alpha_1\gamma_1/(\beta_1\mu_1K_1)$) were changed in a way that kept ρ_{Gal1} invariant. For example, K_1 was divided by 2 and β_1 was doubled (red). (**b-c**) Same analysis but where constituents of ρ_{Gal3} (b) or ρ_{Gal80} (c) were changed (similar formula). (**d**) Same analysis as in b but at lower galactose concentration. All simulations were run with ρ_{Gal1}=100, ρ_{Gal3}=100 and ρ_{Ga80}=250.

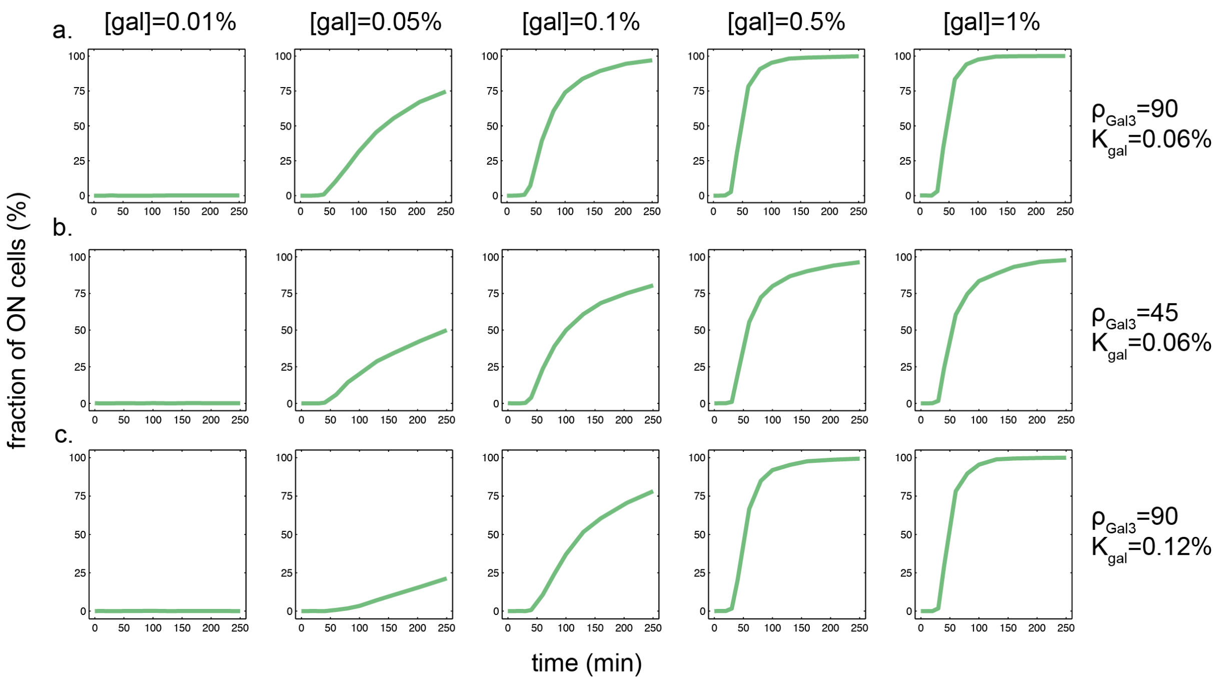

Appendix Figure S5. The network behaviour depends on galactose concentration and on two model parameters. Predictions of the model for the inducibility as a function of time at 5 different galactose concentrations for different values of the GAL3-dependent parameters ρ_{Gal3} and K_{Gal}. GAL3-independent parameters were fixed (Appendix Table S1) with ρ_{Gal1}=100 and ρ_{Gal80}=250 (see main text and Appendix Text S1 for parameter definitions).

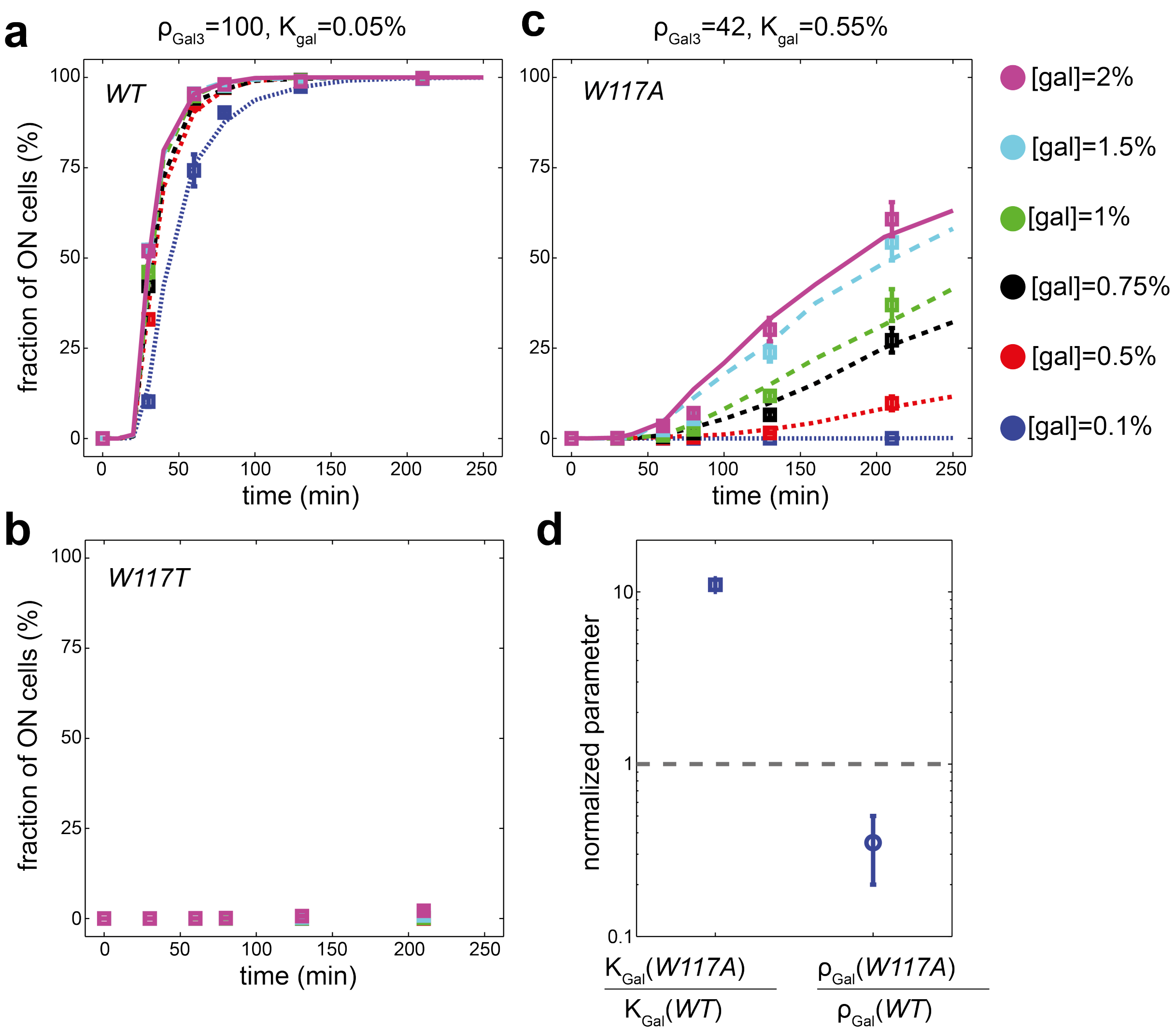

Appendix Figure S6. Effect of W117 mutations of *GAL3*. (**a-c**) Inducibility was measured by flow cytometry (data points +/- s.e.m.) after stimulating cells with different concentrations of galactose (colors). For the wild-type (*WT*, $GAL3^{BY}$) strain and for the *W117A* mutant strain, this data was used to fit the GAL3-dependent parameters ρ_{Gal3} and K_{Gal}. Inferred parameter values are shown. Lines represent the inducibility predicted by the model. (**d**) Ratio of inferred parameter values between the W117A and the WT strain. Inference was performed in the context of 6 different sets of parameters related to GAL1 and GAL80. Bars: standard deviation of the 6 inferred values.

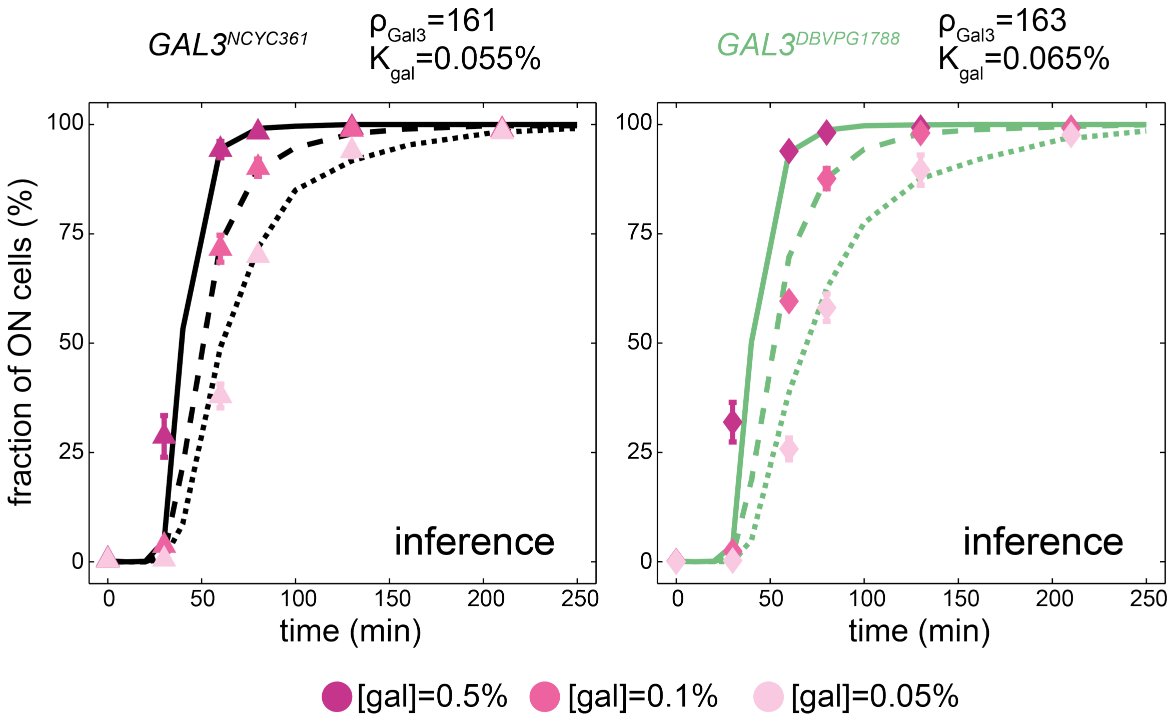

Appendix Figure S7. **Inference of GAL3-dependent model parameters for *GAL3*NCYC361 and *GAL3*DBVPG1788.** Experimentally-measured inducibility of *GAL3*NCYC361 and *GAL3*DBVPG1788 strains, as a function of time, at 3 different galactose concentrations (symbols coloured according to the concentration). These data were used to fit the GAL3-dependent parameters ρ_{Gal3} and K_{Gal}. Full lines (resp. dashed and dotted lines) represent the behaviours predicted by the model at [gal]=0.5% (resp. 0.1% and 0.05%).

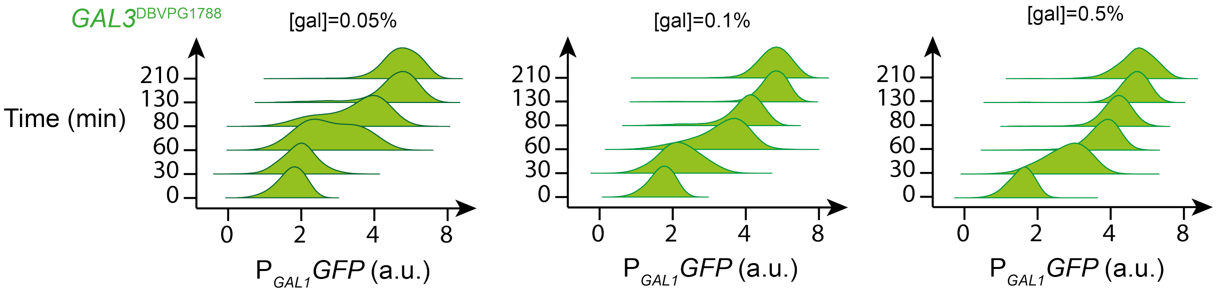

Appendix Figure S8. Time-course flow cytometry data of the *GAL3*^{DBVPG1788} strain, showing its transient binary response at low concentration of inducer (left) and its gradual response at higher concentration (right).

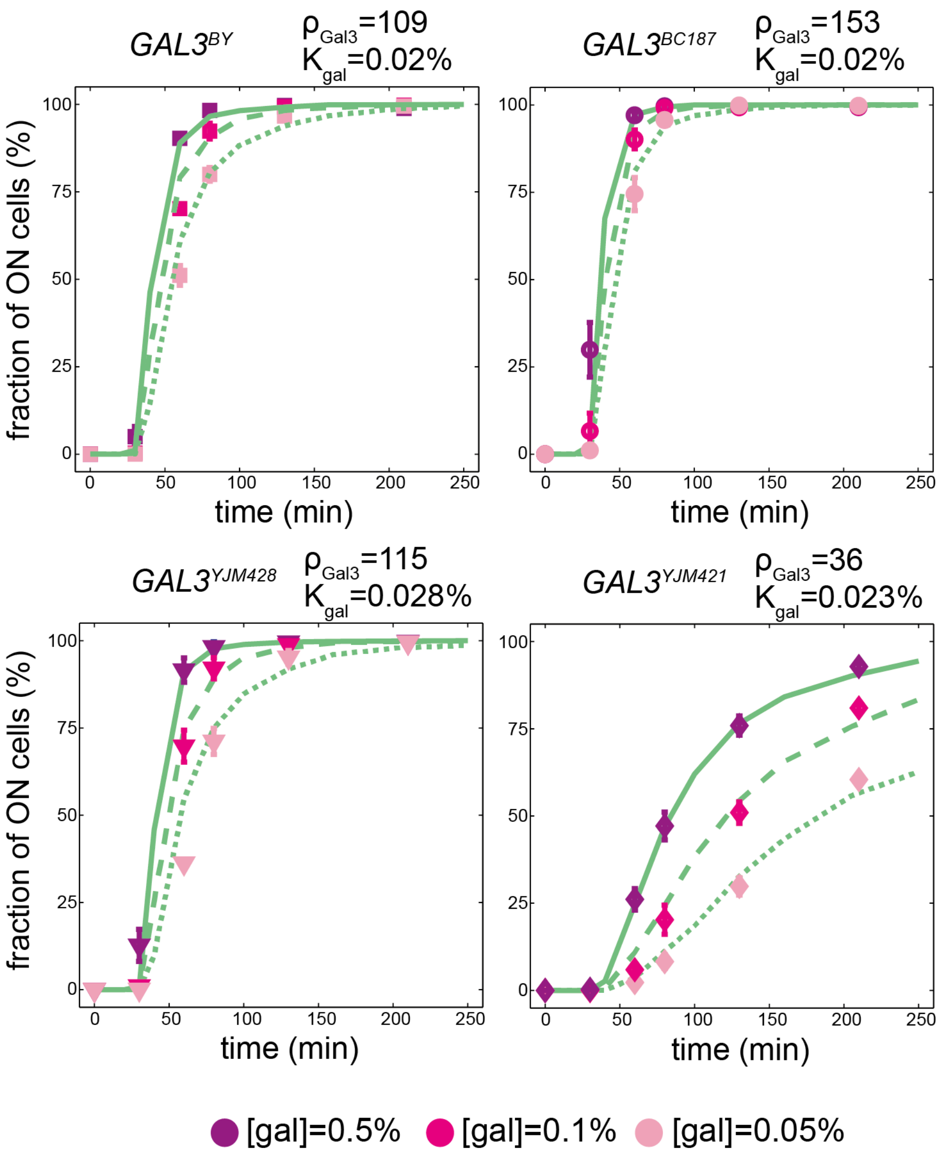

Appendix Figure S9. **Inference of GAL3-dependent model parameters for alleles tested in diauxic shift experiments.** Experimentally-measured inducibility of $GAL3^{BY}$, $GAL3^{YJM421}$, $GAL3^{YJM428}$ and $GAL3^{BC187}$ strains, as a function of time, at 3 different galactose concentrations (symbols coloured according to the concentration). These data were used to fit the GAL3-dependent parameters ρ_{Gal3} and K_{Gal}. Full lines (resp. dashed and dotted lines) represent the behaviours predicted by the model at [gal]=0.5% (resp. 0.1% and 0.05%).

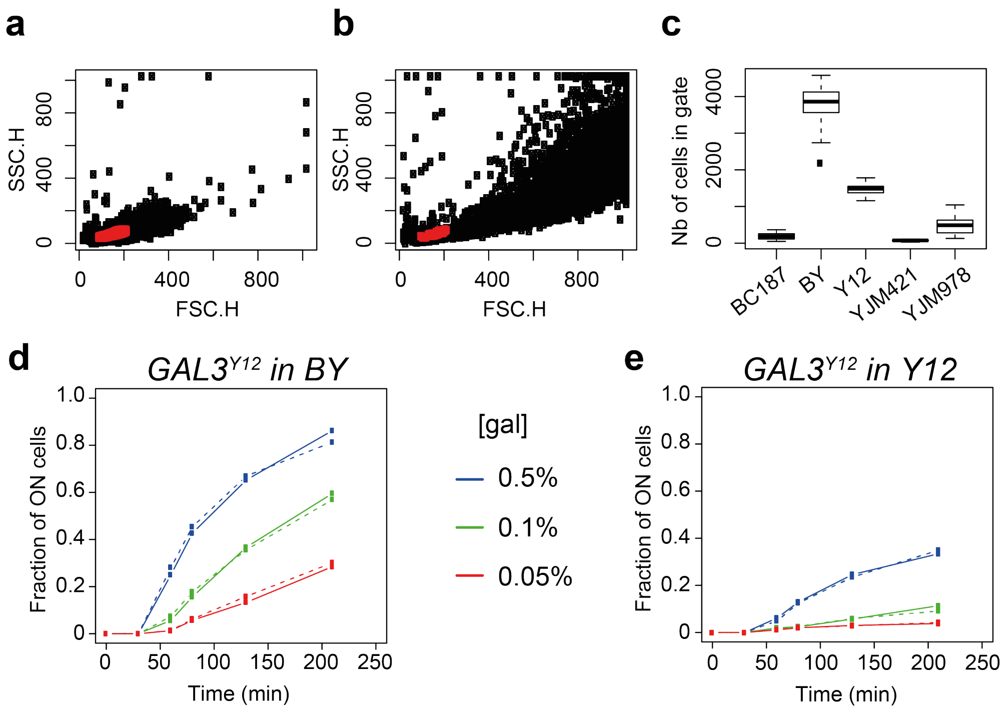

Appendix Figure S10. **Natural variation in *GAL3* alone does not explain the induction dynamics of its original strain.** Strains derived from five distinct natural genetic backgrounds and carrying a P_{GAL1}-YFP reporter were processed through raffinose pre-culture, induction with galactose and time-course flow-cytometry. **a**) A gate was automatically defined on the FSC/SSC channels that contained 40% of events (red dots) in one sample of the BY reference background. **b**) Data of a sample from a strain with YJM421 background. Only few cells (red) fell within the gate. **c**) Number of cells falling in the gate, for all samples grouped by the indicated genetic background. Only the Y12 background provided enough gated cells for comparison to BY. **d-e**) Dynamics of activation of P_{GAL1}YFP when the $GAL3^{Y12}$ allele was in the context of the BY reference background (d) or in its original Y12 context (e).

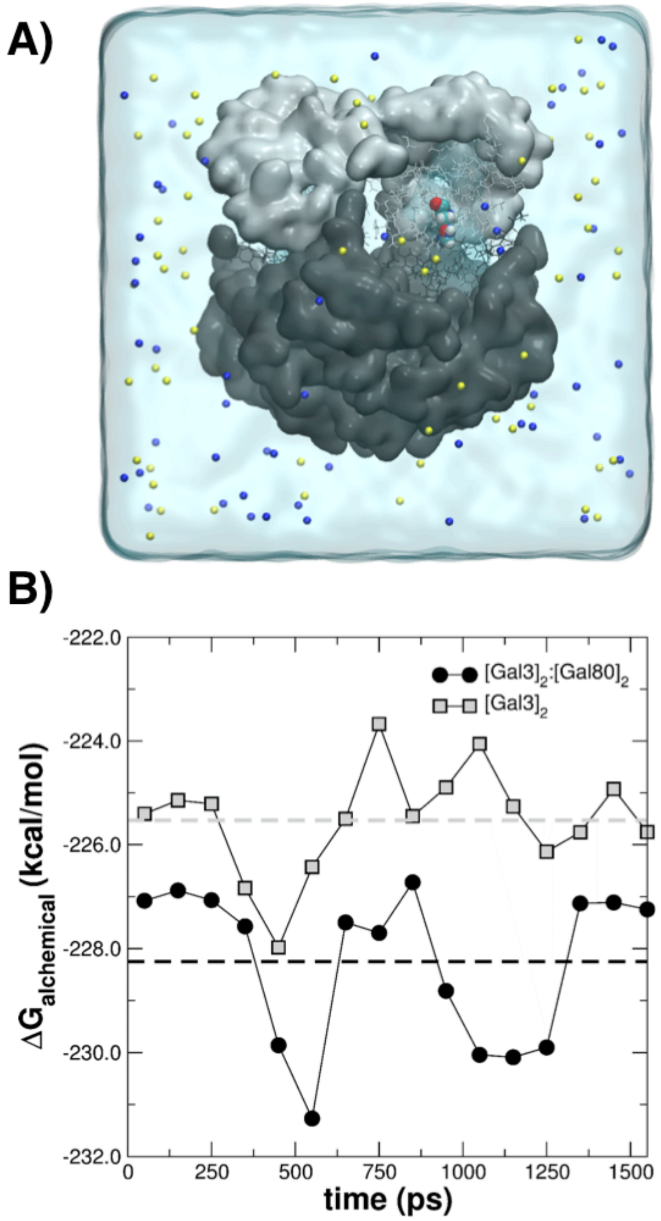

Appendix Figure S11. **Alchemical free energy calculations. A**) Model system for the Gal3p*-Gal80p tetramer used for the alchemical free energy calculations. Residue 352 (of Gal3p*) in the center is shown in colored beads (only one of two possible residues is seen in the chosen orientation). Residues within 15 Å of the two residues 352 were allowed to move freely; they are shown as thin sticks in white (Gal3p*) or grey (Gal80p). Residues that were harmonically restrained are shown as solid surface. This protein substructure was solvated in a cubic box (side length = 90 Å) of water molecules (transparent blueish cube) and salt (blue and yellow dots) with an ionic strength of 0.15 M. **B**) Block analysis of the alchemical free energy calculations. $\Delta G_{alchemical}$ corresponds to the free energy change for transforming 2 x Gal3p*-Asp352 to 2 x Gal3p*-His352. (*i.e.*, changing the coupling parameter λ from 1 to 0, see Appendix Text S2). $\Delta G_{alchemical}$ for the Gal3p* dimer (grey squares) and the Gal3p*-Gal80p tetramer (black circles) is plotted for consecutive blocks of 100 ps of sampling. The horizontal dashed lines indicate the mean values.

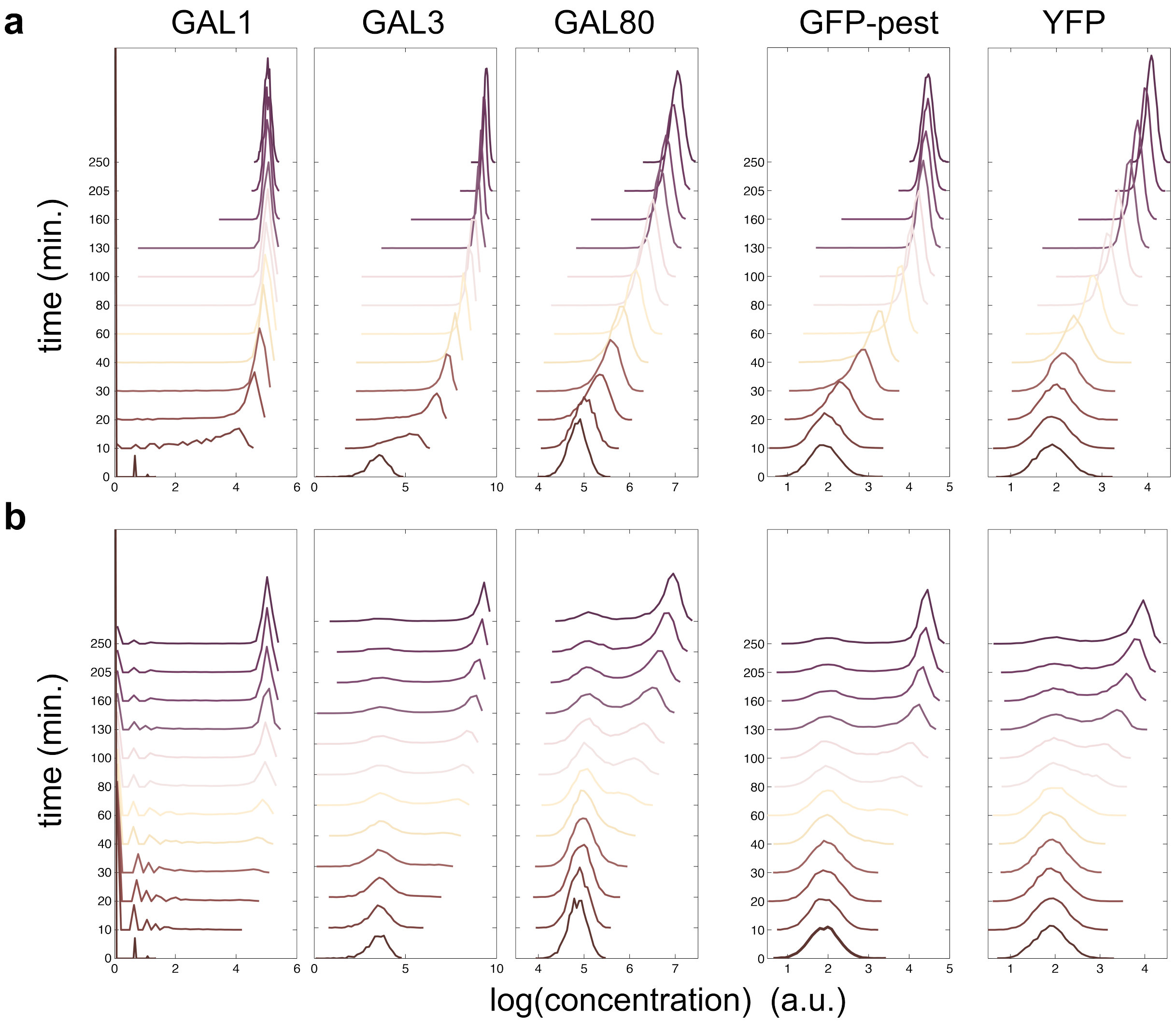

Appendix Figure S12. **The pGal1-GFP induction reflects pathway activity.** Predicted distributions of protein concentration at different time points after pathway induction. (**a**) Using *[gal]=0.5%* and parameters $K_{gal}=0.06\%$ and $\rho_{Gal3}=160$ which match the $GAL3^{NCYC361}$ allele. (**b**) Using *[gal]=0.05%* and parameters $K_{gal}=0.06\%$, $\rho_{Gal3}=90$ which match the $GAL3^{BY}$ allele. The response type (gradual in A and binary in B) is consistent for Gal1p, Gal3p, Gal80p, GFPpest (half-life 30 min) and YFP (half-life 140 min).

**Name**	**Bckg**[(i)]	**Genotype**	**Source**
BY4711	BY	*MATalpha trp1Δ63*	Brachmann et al.
GY1566	BY	*MATa lys2Δ0 ura3Δ0 HIS3:Pgal1-GFPPest-NatMX:HIS3* $GAL3^{BY}$	Chuffart et al. (Chuffart *et al*, 2016)
GY1648	BY	*MATalpha trp1Δ63 HIS3:Pgal1-GFPPest-NatMX:HIS3* $GAL3^{BY}$	*This study*
GY1649	BY	*MATalpha trp1Δ63 HIS3:Pgal1-GFPPest-NatMX:HIS3* $GAL3^{BY}$	*This study*
GY1689	BY	*MATalpha HIS3:Pgal1-GFPPest-NatMX:HIS3* $GAL3^{NCYC361}$	*This study*
GY1692	BY	*MATalpha HIS3:Pgal1-GFPPest-NatMX:HIS3* $GAL3^{K11}$	*This study*
GY1695	BY	*MATalpha HIS3:Pgal1-GFPPest-NatMX:HIS3* $GAL3^{Y12}$	*This study*
GY1698	BY	*MATalpha HIS3:Pgal1-GFPPest-NatMX:HIS3* $GAL3^{DBVPG1788}$	*This study*
GY1704	BY	*MATalpha HIS3:Pgal1-GFPPest-NatMX:HIS3* $GAL3^{DBVPG1853}$	*This study*
GY1707	BY	*MATalpha HIS3:Pgal1-GFPPest-NatMX:HIS3* $GAL3^{YJM978}$	*This study*
GY1713	BY	*MATalpha HIS3:Pgal1-GFPPest-NatMX:HIS3* $GAL3^{JAY291}$	*This study*
GY2009	BY	*MATalpha HIS3:Pgal1-GFPPest-NatMX:HIS3* $GAL3^{BY\text{-}H352D}$	*This study*
GY2180	BY	*MATa lys2Δ0 ura3Δ0 HIS3:Pgal1-GFPPest-NatMX:HIS3* $GAL3^{BY\text{-}W117A}$	*This study*
GY2181	BY	*MATa lys2Δ0 ura3Δ0 HIS3:Pgal1-GFPPest-NatMX:HIS3* $GAL3^{BY\text{-}W117T}$	*This study*
MPJ125-A07	BY	*MATa hoΔ::Gal1pr-YFP-TDH3pr-BFP-NatMX4* $GAL3^{BC187}$	Lee et al. (Lee *et al*, 2017)
MPJ125-E06	BY	*MATa hoΔ::Gal1pr-YFP-TDH3pr-BFP-NatMX4* $GAL3^{S288c}$	Lee et al. (Lee *et al*, 2017)
MPJ143-F01	BY	*MATa hoΔ::Gal1pr-YFP-TDH3pr-BFP-kanMX4* $GAL3^{JYM421}$	Lee et al. (Lee *et al*, 2017)
MPJ143-H01	BY	*MATa hoΔ::Gal1pr-YFP-TDH3pr-BFP-kanMX4* $GAL3^{YJM428}$	Lee et al. (Lee *et al*, 2017)
SLL16-E06	Y12	*MATa hoΔ::Gal1pr-YFP-TDH3pr-BFP-kanMX4* $GAL3^{Y12}$	Lee et al. (Lee *et al*, 2017)
SLL16-B06	BC187	*MATa hoΔ::Gal1pr-YFP-TDH3pr-BFP-kanMX4* $GAL3^{BC187}$	Lee et al. (Lee *et al*, 2017)
SLL16-G06	YJM978	*MATa hoΔ::Gal1pr-YFP-TDH3pr-BFP-kanMX4* $GAL3^{YJM978}$	Lee et al. (Lee *et al*, 2017)
SLL16-H06	YJM421	*MATa hoΔ::Gal1pr-YFP-TDH3pr-BFP-kanMX4* $GAL3^{YJM421}$	Lee et al. (Lee *et al*, 2017)

(i): General genetic background

Appendix Table S3: Strains used in this study.

ID	Sequence (5' to 3')
1D28	AGAGGCGGTGGAGATATTCCTTATG
1D56	ACGTCCGCTATACCTTCGTTTTCTC
1M95	tctttcattatgtgagagtttaaaaaccagaaactacatcatcgaaaaagggatccAGAGGCGGTGGAGATATTCCTTATG
1M96	cgcccaatacgcaaaccgcctctccccgcgcgttggccgattcattaatgcagctgACGTCCGCTATACCTTCGTTTTCTC
1J63	CCGCGCAAGTAAATGCAGATGA
1P21	TGATTTTATCGCTAGGTCTC
1P22	AAAGTTTGATCTGCCTTTAGATGGTTCCTACATGGCCATAGATCCGTCTGTGTCGGAAgcgTCGAATTACTTTAAATGC
1P23	AAAGTTTGATCTGCCTTTAGATGGTTCCTACATGGCCATAGATCCGTCTGTGTCGGAAacgTCGAATTACTTTAAATGC
1P29	GATCTAGATCCGTCTGTGTCGGAAGTTTTAGAGCTAG
1P30	CTAGCTCTAAAACTTCCGACACAGACGGATCTA

Appendix Table S4: DNA primers used in this study

REFERENCES

Acar M, Becskei A & van Oudenaarden A (2005) Enhancement of cellular memory by reducing stochastic transitions. *Nature* **435:** 228–32

Apostu R & Mackey MC (2012) Mathematical model of GAL regulon dynamics in Saccharomyces cerevisiae. *J Theor Biol* **293:** 219–35

Archontis G, Simonson T, Moras D & Karplus M (1998) Specific amino acid recognition by aspartyl-tRNA synthetase studied by free energy simulations11A. R. Fersht. *J. Mol. Biol.* **275:** 823–846

Baker NA, Sept D, Joseph S, Holst MJ & McCammon JA (2001) Electrostatics of nanosystems: Application to microtubules and the ribosome. *Proc. Natl. Acad. Sci.* **98:** 10037–10041

Bar-Even A, Paulsson J, Maheshri N, Carmi M, O'Shea E, Pilpel Y & Barkai N (2006) Noise in protein expression scales with natural protein abundance. *Nat Genet* **38:** 636–43

Bennett CH (1976) Efficient estimation of free energy differences from Monte Carlo data. *J. Comput. Phys.* **22:** 245–268

Brooks BR, Brooks CL, Mackerell AD, Nilsson L, Petrella RJ, Roux B, Won Y, Archontis G, Bartels C, Boresch S, Caflisch A, Caves L, Cui Q, Dinner AR, Feig M, Fischer S, Gao J, Hodoscek M, Im W, Kuczera K, et al (2009) CHARMM: The biomolecular simulation program. *J. Comput. Chem.* **30:** 1545–1614

Chipot C & Pohorille A (2007) Calculating Free Energy Differences Using Perturbation Theory

Chuffart F, Richard M, Jost D, Burny C, Duplus-Bottin H, Ohya Y & Yvert G (2016) Exploiting Single-Cell Quantitative Data to Map Genetic Variants Having Probabilistic Effects. *PLoS Genet.* **12:** e1006213

Dolinsky TJ, Nielsen JE, McCammon JA & Baker NA (2004) PDB2PQR: an automated pipeline for the setup of Poisson–Boltzmann electrostatics calculations. *Nucleic Acids Res.* **32:** W665–W667

Figueirido F, Del Buono GS & Levy RM (1997) On Finite-Size Corrections to the Free Energy of Ionic Hydration. *J. Phys. Chem. B* **101:** 5622–5623

Gasch AP, Spellman PT, Kao CM, Carmel-Harel O, Eisen MB, Storz G, Botstein D & Brown PO (2000) Genomic expression programs in the response of yeast cells to environmental changes. *Mol Biol Cell* **11:** 4241–57

Gillespie DT (1977) Exact stochastic simulation of coupled chemical reactions. *J. Phys. Chem.* **81:** 2340–2361

Giniger E & Ptashne M (1988) Cooperative DNA binding of the yeast transcriptional activator GAL4. *Proc. Natl. Acad. Sci. U. S. A.* **85:** 382–386

Höchtl P, Boresch S, Bitomsky W & Steinhauser O (1998) Rationalization of the dielectric properties of common three-site water models in terms of their force field parameters. *J. Chem. Phys.* **109:** 4927–4937

Hsu C, Scherrer S, Buetti-Dinh A, Ratna P, Pizzolato J, Jaquet V & Becskei A (2012) Stochastic signalling rewires the interaction map of a multiple feedback network during yeast evolution. *Nat. Commun.* **3:** 682

Hummer G, Pratt LR & García AE (1996) Free Energy of Ionic Hydration. *J. Phys. Chem.* **100:** 1206–1215

Humphrey W, Dalke A & Schulten K (1996) VMD: Visual molecular dynamics. *J. Mol. Graph.* **14:** 33–38

Iizuka R, Yamagishi-Shirasaki M & Funatsu T (2011) Kinetic study of de novo chromophore maturation of fluorescent proteins. *Anal. Biochem.* **414:** 173–178

Lavy T, Kumar PR, He H & Joshua-Tor L (2012) The Gal3p transducer of the GAL regulon interacts with the Gal80p repressor in its ligand-induced closed conformation. *Genes Dev.* **26:** 294–303

Lee J, Cheng X, Swails JM, Yeom MS, Eastman PK, Lemkul JA, Wei S, Buckner J, Jeong JC, Qi Y, Jo S, Pande VS, Case DA, Brooks CL, MacKerell AD, Klauda JB & Im W (2016) CHARMM-GUI Input Generator for NAMD, GROMACS, AMBER, OpenMM, and CHARMM/OpenMM Simulations Using the CHARMM36 Additive Force Field. *J. Chem. Theory Comput.* **12:** 405–413

Lee KB, Wang J, Palme J, Escalante-Chong R, Hua B & Springer M (2017) Polymorphisms in the yeast galactose sensor underlie a natural continuum of nutrient-decision phenotypes. *PLOS Genet.* **13:** e1006766

Lin M-T, Wang C-Y, Xie H-J, Cheung CHY, Hsieh C-H, Juan H-F, Chen B-S & Lin C (2016) Novel Utilization of Terminators in the Design of Biologically Adjustable Synthetic Filters. *ACS Synth. Biol.* **5:** 365–374

Mateus C & Avery SV (2000) Destabilized green fluorescent protein for monitoring dynamic changes in yeast gene expression with flow cytometry. *Yeast* **16:** 1313–23

Melcher K & Xu HE (2001) Gal80-Gal80 interaction on adjacent Gal4p binding sites is required for complete GAL gene repression. *EMBO J.* **20:** 841–851

Milo R, Jorgensen P, Moran U, Weber G & Springer M (2010) BioNumbers—the database of key numbers in molecular and cell biology. *Nucleic Acids Res.* **38:** D750–D753

Paulo JA, O'Connell JD, Gaun A & Gygi SP (2015) Proteome-wide quantitative multiplexed profiling of protein expression: carbon-source dependency in Saccharomyces cerevisiae. *Mol. Biol. Cell* **26:** 4063–4074

Phillips JC, Braun R, Wang W, Gumbart J, Tajkhorshid E, Villa E, Chipot C, Skeel RD, Kalé L & Schulten K (2005) Scalable molecular dynamics with NAMD. *J. Comput. Chem.* **26:** 1781–1802

Pilauri V, Bewley M, Diep C & Hopper J (2005) Gal80 Dimerization and the Yeast GAL Gene Switch. *Genetics* **169:** 1903–1914

Song R, Peng W, Liu P & Acar M (2015) A cell size- and cell cycle-aware stochastic model for predicting time-dynamic gene network activity in individual cells. *BMC Syst. Biol.* **9:** 91

Timson DJ, Ross HC & Reece RJ (2002) Gal3p and Gal1p interact with the transcriptional repressor Gal80p to form a complex of 1:1 stoichiometry. *Biochem. J.* **363:** 515–520

Tschopp JF, Emr SD, Field C & Schekman R (1986) GAL2 codes for a membrane-bound subunit of the galactose permease in Saccharomyces cerevisiae. *J. Bacteriol.* **166:** 313–318

Venturelli OS, El-Samad H & Murray RM (2012) Synergistic dual positive feedback loops established by molecular sequestration generate robust bimodal response. *Proc. Natl. Acad. Sci.* **109:** E3324–E3333
